# Some Advice for Physicians and Other Clinicians Treating Minorities, Women, and Other Patients at Risk of Receiving Health Care Disparities

**DOI:** 10.1007/s40615-016-0248-6

**Published:** 2016-06-10

**Authors:** Augustus A. White, Beauregard Stubblefield-Tave

**Affiliations:** 1 Harvard Medical School, Boston, MA USA; 2 The Center for Culturally Fluent Leadership, Newton, MA USA

**Keywords:** Health care, Health disparities, Health equity, Cultural competence, Cultural humility, Unconscious bias, Minority health, Women’s health, Patient clinician relationship, Patient empowerment, Conscious bias, Microaggressions, Implicit bias, Equitable care

## Abstract

Studies of inequalities in health care have documented 13 groups of patients who receive disparate care. Disparities are partly due to socioeconomic factors, but nonsocioeconomic factors also play a large contributory role. This article reviews nonsocioeconomic factors, including unconscious bias, stereotyping, racism, gender bias, and limited English proficiency. The authors discuss the clinician’s role in addressing these factors and reducing their impact on the quality of health care. They indicate the significance of cultural humility on the part of caregivers as a means of amelioration. Based on a review of the clinician’s role as well as background considerations in the health care environment, the authors put forward a set of 18 recommendations in the form of a checklist. They posit that implementing these recommendations as part of the patient clinician interaction will maximize the delivery of equitable care, even in the absence of desirable in-depth cross-cultural and psychosocial literacy on the part of the clinician. Trust, mutual respect, and understanding on the part of the caregiver and patient are crucial to optimizing therapeutic outcomes. The guidelines incorporated here are tools to furthering this goal.

“The good physician treats the disease; the great physician treats the patient who has the disease*.*” -Sir William Osler

## Introduction

Health care organizations shall “provide effective, equitable, understandable, and respectful quality care and services that are responsive to diverse cultural health beliefs and practices, preferred languages, health literacy, and other communication needs.” This statement is from the National Standards for Culturally and Linguistically Appropriate Services (CLAS) [1]. This can be thought of as depicting a kind of a “gold standard” by which to define a desirable quality of care.

Clinicians are trained in the art and science of providing health care. Caregivers have the privilege of partnering with patients^1^ to cure disease or injury and promote well-being. In every encounter, the intent is to “provide effective, equitable, understandable, and respectful care and services.” [1] Nevertheless, the Institute of Medicine (IOM) found that “racial and ethnic minorities tend to receive a lower quality of health care than non-minorities” [2] which means that some of us are delivering a lower quality of health care to certain groups*.* The IOM’s finding of disparate care applies to a number of component segments of American society including, but not limited to:

1. Women
2. African Americans
3. Appalachian poor
4. Asian Americans
5. Elders
6. Immigrants and refugees^2^ [3]
7. Individuals living with disabilities
8. Latinos/Hispanics
9. Members of the lesbian, gay, bisexual, and transgender, questioning, intersex, and asexual community
10. Native Americans.
11. Overweight people
12. Prisoners
13. Some religious minorities

The sources of health and health care disparities are “complex and involve many participants at several levels.” [2] This paper focuses on the level of the individual clinical encounter—where it is you, the clinician, and your partner, the patient, battling against common enemies: disease and poor health. There are factors in every patient clinician encounter that can lead to unequal treatment; however, there are measures you can take to combat those factors.

## Background Considerations: Factors that Potentiate Unequal Treatment in the Clinical Encounter

### Unconscious Bias

It is known that unconscious bias is an ever present and powerful reality affecting many of our day-to-day decisions. Physicians are not immune to unconscious bias. “Moreover, we know that the likelihood of unconscious or subconscious factors affecting decision making is much greater for individuals under stress and/or doing multi-tasking [2],” a description that fits physicians and other clinicians especially well.

### Stereotyping

“People have a propensity to stereotype; it is a universal human trait, and often damaging or dangerous for those who are stereotyped.” [4].

Kevin Schulman dramatically demonstrated the potential impact of unconscious bias on patient care by presenting physicians with simulated patients who had identical cardiac symptoms. The only differences among the patients were race and gender. Black women were significantly less likely than any other group to be recommended for cardiac catheterization, the appropriate treatment for the symptoms presented. “If you were black, the report concluded, you were less likely to be referred for catheterization. If you were a woman, you were also less likely to be referred. And if you were a black woman, you were especially less likely to be referred*.*” [5].

### Gender Bias in Clinical Care

Women of all backgrounds are subject to unequal treatment, and women of color are especially subject to inferior care. Consider this example: “Doctors were more likely to believe that the heart problems of women who reported stress were due to psychological causes, whereas men who talked about stress were considered more likely to have organic heart disease.” [6] Women consistently receive less in the way of aspirin, beta-blockers, and cholesterol-lowering drugs after they have had a heart attack. Researchers have also found that it can take significantly longer for emergency medical personnel to get women to the hospital with heart attacks [7].

### Racism: Institutionalized, Ambient, and Internalized

Camara Phyllis Jones defined institutionalized racism as “differential access to the goods, services, and opportunities of society by race.” [8] Institutionalized racism is “structural, having been codified in our institutions of custom, practice, and law, so there need not be an identifiable perpetrator. Indeed, institutionalized racism is often evident as inaction in the face of need.” [8] Ambient racism is reflected in the broader society of which health care is a part. Internalized racism is also damaging to minority health. Jones defines Internalized racism as “acceptance by members of the stigmatized races of negative messages about their own abilities and intrinsic worth.” [8].

Race has proven to be a powerful factor in clinical communications: Physicians were 23 % more verbally dominant in conversation with African American patients than with White patients. Physicians also engaged in 33 % less total conversation with African American patients than with White patients [9].

### Sexual Orientation

Gay men and lesbians have reported receiving demonstrably inferior care for some time [10]. Recent research indicates that bisexuals have worse health outcomes than their heterosexual, gay, or lesbian counterparts [11]. This may be caused, in part, by conscious and unconscious bias: “Societal biphobia — negative attitudes and behaviors toward bisexual individuals — is more prevalent than antigay sentiment.” [11] Further, some bisexuals report feeling like “minorities within a minority” and receive less social support and recognition than gays and lesbians. Consider your own situation. Ask yourself: “Do I have any bisexuals in my practice?” Does one have an accurate answer? One cannot be sure of the answer unless one has asked their patient. Asking every patient their sexual orientation, just as one asks their age, gender, and race, is one part of understanding and accepting “the patient who has the disease.” One also needs to ask oneself, “Do I have an unconscious bias towards sexual minorities?” and respond to your honest answer.

### Limited Health Literacy [12]

Nearly nine out of ten adults may lack the skills needed to manage their health and prevent disease. Please remember that patients with limited literacy may work very hard to hide it. Given these circumstances, testing for understanding, e.g., using the “teach-back” technique, is critical.

### Interpretation Quality [13]

Title VI of the federal Civil Rights Act mandates that patients who speak English “less than very well” have limited English proficiency (LEP) must receive care in their native language. This care must be provided at no charge through bilingual clinicians or qualified interpretation services. “Professional interpreters result in a significantly lower likelihood of errors of potential consequence than clinical encounters with ad hoc and no interpreters*.*”

### Cross-Cultural Communication Challenges

Family Physician Robert Like says, “every clinical encounter is a cross-cultural encounter*.*” [14] The cross-cultural reality is that even a patient who matches her doctor on age, gender, race, and native language will differ on other cultural dimensions. These could be family status, national origin, sexual orientation, and profession.

In this section, the authors have pointed out a substantial number of realities that can make the patient clinician interactions difficult. There are more. In the following contents of this publication, the authors seek to provide ideas and suggestions which it is believed will constitute at least partial solutions to formidable realities of health care disparities, at least as they are manifested in the patient clinician interactions.

## Background Considerations: the Clinician’s Role

The authors respectfully submit to our readers the following ideas. The privilege of professional entitlement and training carries with it responsibility and the need for dedication to certain moral and ethical responsibilities. For each patient or client, one aspires to provide the very best care and to not in any way slight or compromise the care of any individual patient.

While clinicians have the privileges and responsibilities of professionalism, please be reminded that the patient has his or her own moral, ethical, and legal right to expect compassionate care that is not compromised, consciously or unconsciously, by harmful human biases on the part of the clinician.

Commit yourself to answering the patient’s questions with compassion and a commitment to a clear understanding. Achieving such understanding can be challenging, and yet this understanding must be achieved. It may be necessary to provide or direct the patient to supplemental handouts, reading materials, videos, websites, or other resources. The patient can and should be expected to make some effort to understand their condition, treatment alternatives, and other actions that may improve their health. As a clinician, however, one retains the responsibility to assure that our patients understand what has been communicated to them.

Clearly, physicians and other clinicians in the health professions are very engaged in an inordinate number of stressors in our profession at this time. Consider developing specific mechanisms for managing this stress. This is essential to our own well-being and ability to deliver high-quality care.

### Cultural Humility

The clinician’s role is further explored in the concept of “Cultural Humility,” a multi-dimensional concept that offers three central principles: lifelong learning, self-reflection, and self-critique [15]. Lifelong learning and self-reflection include examining one’s own cultural identity, unconscious and conscious biases, and patterns of thought and behavior that improve or detract from effective patient care. How well does one listen to our patients? Does the caliber of our listening vary based on the patient’s race, gender, sexual orientation, et cetera? Does one consistently create a welcoming environment for each patient? Asking such questions, and acting upon our honest reflections, is vital to providing the “exceptional care without exception” that caregivers seek to offer.

### Identifying and Challenging Power Imbalances in the Clinical Relationship

The clinician in every clinical encounter has significant power relative to the patient [15]. Addressing power differentials therefore is a vital aspect of patient care. Such power differentials are magnified when the patient and clinician come from different class backgrounds. For example, conversation regarding drugs was significantly less successful for patients from lower SES backgrounds [16]. Patients from higher socioeconomic background had 60 % more conversation with their physicians [17].

Clinicians must help their patients understand that they must have a voice in the clinical encounter. The goal is to “treat the patient who has the disease*.*” The goal is to care for them as a whole, complex person with a family history and cultural identity that influence their health. Better care can be provided when the patient can be cared for as a person and a patient. Patients should be encouraged to ask questions. Also, patients should be assisted to learn about their disease. These activities will enable them to be more equal and more effective partners in their care. Using patient-focused interviewing techniques, e.g., the “teach-back,” will also help reduce power differentials. All these efforts enhance the partnership of the patient and the clinician against the disease!

### Promoting Institutional Accountability

Tervalon [15] describes the concept of “institutional accountability” as the part of cultural humility that people “do not read or do not like.” The concept requires that organizations perform the same self-examination, lifelong learning, and challenging of power imbalances that clinicians do. Hospitals, health centers, and group practices can use surveys, focus groups, and similar techniques to identify unconscious biases. Providing training to clinicians, patients, and family members can help reduce power imbalances. These efforts can be embedded into ongoing quality improvement efforts to ensure that they have their intended impact and are sustained over time. No individual clinician can hold an entire institution accountable of course. At the same time, every clinician has the ability and responsibility to advocate for their institution’s provision of culturally competent care.

## Advice/Recommendations/“Checklist”

This project of a culturally competent care checklist began with a presumed mindset of originality. Subsequently, the milestone publication [18] which includes in its Appendix A “Checklist to Improve Effective Communication, Cultural Competence, and Patient- and Family- Centered Care Across the Care Continuum” was discovered. This important publication helped and emboldened the authors considerably.

Presented here is a “checklist” of specific, detailed, complex guidelines, some of which are not easy to follow. Few, if any of us, are already actively, skillfully engaged in the successful execution of all 18 items on this checklist. However, the authors are offering the list as a set of guidelines to be used as a quick reference to help us improve and develop our clinical skills so as to be better partners in collaboration with our patients so as to provide the best possible team to fight their disease and promote their health. So clinicians are encouraged to follow as much of the checklist as well as possible, as often as possible as one enjoys improvement going forward.

1. **Wash your hands, of course**.
2. **Humanize your patient**. All of these recommendations are important, yet this may be the most important: Humanize your patient! What does this mean? This means to communicate with the patient in a way that connects your humanness with the patient’s humanness. This involves body language, physical touch, voice inflection, and most important, the content of your communications. Some examples are discussion about family, the weather, an athletic event, spirituality and religion, food, travel, music, and how you met your spouse or significant other. One can simply ask the patient “Is there anything you’d like me to know personally, as a fellow human being, that would help me to give you the best care possible?” Humanizing your patient means building a level of personal relationship with your patient. Some patients will be quite satisfied shaking your hand, while others will want a hug of reassurance after discussing a difficult diagnosis or the loss of a loved one. Your personal, clinical relationship will vary over time based on your preferences and those of your patient. When addressing sensitive topics, e.g., religion, it is useful for the clinician to follow the patient’s lead as to when and how to address the topic. Why is humanizing the patient so important? Experience suggests that seeing a patient as individual human being helps reduce the power of stereotypes and promotes trust in both individuals. Further, patient satisfaction was linked to surgeon empathy [12, 26].
  Both partners can share in humanizing the other. The authors suggest that the patient do a bit of research on you, for example, before you meet. Many organizations create clinician biographies that enable you to describe your clinical philosophy, interests, and hobbies to your patients. Google, Facebook, LinkedIn, and other sites may also provide additional information.
3. **Identify and monitor conscious and unconscious biases**. All human beings have biases. The conscious biases are readily identified. Unconscious biases, by definition, cannot be observed directly. It is reasonable to assume that all of us have some. In the clinical setting, one may receive feedback regarding conscious and/or unconscious bias. This feedback may come from patients, colleagues, or supervisors. Essentially, no caregiver is immune and this situation is not a disaster for anyone dealing with such sensitive issues. Please seek counsel with trusted colleagues.
  “Counteracting unconscious bias requires awareness, introspection, authenticity, humility, compassion, communication and a willingness to act.” Tools such as the Implicit Association Test [19] can raise our awareness of specific forms of unconscious bias. The website provides 14 rigorous tests that are available free of charge. They include tests regarding age, disability, race, and sexuality. Once biases are identified and there is motivation to change them, what actions can be taken?
  Explore the history and cultural traditions of populations being served, and discuss experiences with patients from the various cultural backgrounds involved. Some patients appreciate this interest and share information readily. This humanizes the patient, reduces power differentials, and counteracts unconscious bias through engagement with individuals who do not fit biased images of them.
  Diversity expert Howard Ross [20] recommends several steps to identify and address unconscious bias. Recognize that one has biases. Identify what those biases are. Dissect (explore) those biases. Decide which biases should be addressed first.
4. **Do a teach-back**. A teach-back is comprised of the following exercise: Before the visit is over, ask the patient to describe, in their own words, what they understand their health problem to be and what is being done to address the problem. If the patient understands both, congratulate yourself and the patient. If not, respectfully correct the inaccuracies or lack of completeness that comprise the patient’s answer, then again ask the patient to respond to the same questions “What do you understand to be the problem? What is being done to solve it?” If it is correct this time, congratulate the patient. If it is not, repeat the process and continue until the answer is satisfactory. Yes, this is time consuming, yet one becomes more efficient with practice. Further, consider how the benefits of clear, compassionate communication compares with the expenditure of time and costs expended when there is miscommunication.
5. **Help the patient to learn about his or her disease or condition**. Patient education is one of the most powerful tools in any clinician’s toolkit. *Web MD*, the Mayo Clinic Portal, and websites provided by the federal government are examples of peer-reviewed sources of medical information clinicians can recommend to their patients. Some health care organizations provide live and online courses on common health issues from Alzheimer’s and diabetes to smoking cessation and weight management [21]. In addition, support groups offer excellent education online and in person.
6. **Welcome a patient’s friend, partner, and/or family members**. The patient may well have been encouraged to bring along a friend or relative. If that is the case, please welcome and acknowledge those individuals. If the clinician determines it is desirable to instead meet with the patient alone, the clinician should carefully explain their request and the reason for this change to both the visitor and the patient. A respectful inquiry about the relationship with the friend, if not clear, may provide information and insight and improve the clinical encounter and the relationship with the patient. This could provide a comfortable opportunity for patient or caregiver to discuss information about sexual orientation and/or gender identity. If the patient does not have a support person, suggest that they arrange to have one.
7. **Learn a few key words and phrases in the most common languages in your area**. A dentist once reported learning key phrases in 15 languages. These included, “hello,” “thank you,” “open wide,” and “does this hurt?” The dentist demonstrated a degree of compassion, a willingness to learn, and a welcoming attitude that many patients appreciated. For most clinicians, words from two to three languages would make a major impact.
8. **Use a qualified medical interpreter as appropriate**. If there is any doubt that the patient is experiencing limited English proficiency, it is the responsibility of the caregiver to utilize the expertise of a qualified medical interpreter. Under Title VI of the Civil Rights Act, such an interpreter must be provided at no charge to the patient.
9. **Be aware of the potential for “false fluency**.” [22]—If the caregiver is communicating with patients in a language that is not one’s native language, the caregiver’s language skills should be tested and certified prior to communicating with the patients in that language in the treatment process [22].
10. **Seek training in working with an interpreter**. A brief training in working with an interpreter can be very valuable in caring for one’s patients^3^,^4^ [22, 23]. For example, one would be taught to speak directly to the patient as though the interpreter is not there. The interpreter acts as a linguistic conduit: repeating everything that is being said to the patient in the patient’s language, repeating everything the patient says to the caregiver in English. With your permission, some interpreters will also act as a “cultural broker.”^5^ In this role, the interpreter will explain concepts that exist in the patient’s culture that do not exist in American culture.
11. **Consider the health literacy of one’s patients**. Be alert for someone for whom English is their native language, but who may have literacy considerations that could greatly limit communications. For example, some patients may be unable to read and follow written instructions on a medication label. The patient may be embarrassed by this fact and may not readily admit this; nevertheless, understanding is critical. One may use visual guides to promote understanding and adherence.
12. **Know the patients’ rights and responsibilities**. All patients have specific rights outlined in federal law and policy. Many states, hospitals, health plans, and others describe additional rights. In some cases, they also identify patient responsibilities. Patients may not be aware of these rights and responsibilities; nevertheless, both the caregiver and the patient should be. The caregiver is an advocate for and an educator of the patient. Here is a reference that provides a summary of patients’ rights and responsibilities [13]. Patients expect the caregiver to help them improve their health and advocating for their rights is a part of the role as a clinician. The authors recommend a review of this publication on Medical Professionalism [24].
13. **Encourage the patients to ask questions**. Many Americans today are eager to ask their clinicians about medications they have seen advertised on TV or treatments they have read about on the Internet. Clinicians can get frustrated with such questions, particularly when they are based on marketing interests rather than scientific research. Nevertheless, the authors strongly recommend that patients be encouraged to ask questions. The patient will generally know more than the caregiver about their own health as they experience it. When the patients’ questions are viewed as part of the continuing medical education, this can be valuable to both patient and clinician.
14. **Respond thoughtfully to patient complaints**. If the patient should complain directly about the care they are receiving during the visit, the following response is suggested. First, apologize. Do not make any excuse, but ask the necessary questions to be sure to understand the complaint. The complaint may be that inequitable care is being provided, or the patient believes that the caregiver is acting, based on some personal bias or prejudice based on the patient’s gender, race, ethnicity, or other possible target of bias. Indicate that the motivation, desire, and intent is to provide the medical care that the patient has come to seek and deserves. Ask if the caregiver and patient can work together and go forward in a collegial partnership. That partnership would involve the clinician and the patient working together to successfully confront the disease or condition as a team. Do this forthrightly and respectfully. The authors believe that the patient will usually respond affirmatively, if so, the situation is likely resolved and the patient and caregiver can proceed together. Suppose the patient does not believe such a partnership is possible. Respect the patient’s decision, of course. Also, one may wish to offer assistance in finding an alternative clinician.
15. **Discuss a second opinion**. If consideration of elective surgery is part of the clinical picture, and there is the anxiety, stress, worry, or misunderstanding about this, consider asking the patient if they would like a second opinion. If the patient needs surgery and is very reluctant to have it, a second opinion may help, or if the patient does not need surgery, and is adamant about the idea that he or she does need surgery, again, a second opinion can be helpful.
  Second opinions can also be helpful when patients face alternative treatments: surgery, chemotherapy, radiation, or some combination to treat cancer, for example. Presenting the possibility of a second opinion can reassure a patient that the clinician is focused on their health, rather than on the caregiver’s personal gain.
16. **Hold one’s institutions accountable for providing culturally and linguistically competent care**. As an individual clinician, one has a limited, yet vital ability to hold affiliated institutions (hospitals, group practices, health plans) responsible for demonstrating cultural humility and providing culturally and linguistically competent care. The federal CLAS Standards, Joint Commission accreditation standards, and the caregiver’s own institutional by-laws and protocols may be helpful [25].
  The seminal article “Medical Professionalism in the New Millennium” asserts that physicians have a professional commitment not just to individual patient care, but also to address societal factors that affect the health of patients. Such improvement requires physicians to participate in or be aware of assessment of “the performance of all individuals, institutions, and systems responsible for health care delivery.” [24].
17. **Advocate that the affiliated institution’s analyses of patient satisfaction and outcome include cultural group data and that the results lead to concrete action**. The vast majority of provider organizations collect some form of patient satisfaction and clinical outcome data. Do affiliated institutions analyze the data by cultural groups? Do they identify and respond to disparities? Political will is required to conduct such analysis, and particularly, to respond to the results. Clinicians have the ability and the responsibility to advocate for such work on behalf of patients.
18. **Encourage patients to complete patient satisfaction and demographics forms**. Some patients choose to avoid patient satisfaction and demographics forms because they see them as a waste of time; others see them as an invasion of privacy. Still others believe that the data they share will be used against them by the provider organization, Immigration and Customs Enforcement or the National Security Agency. Patient data is critical to quality improvement and disparities reduction. Provider organizations work very hard to assure the confidentiality of patient data and to analyze such data without identifying any individual’s responses. Clinicians have the patient’s best interests at heart and some degree of credibility with them. Please encourage patients to take the time to fill out these forms and explain to them how the data will be used. In order to save clinician time, it is reasonable to train someone else in the registration chain to do this.

## Concluding Comments

There are 18 points on this checklist. There is neither time nor necessity to apply them all to each patient. With experience, the caregiver will select more appropriately and execute the checklist items more effectively.

Caregivers cannot control health policy for the nation, and possibly not even in the local health facilities. Clinicians cannot control the health insurance industry. Clinicians cannot control other doctors, nurses, or staff members in health facilities. Caregivers can control themselves as individuals! It is important to recognize the stressors affecting clinicians and to take precautions to develop ways of adjusting and coping with those stressors. Recognize that even under the best of circumstances, there will be some rough spots in interacting with patients. Patient care is an extremely difficult and challenging activity. Caregivers must not neglect their own physical and emotional health as they assiduously address the formidable challenges of providing equitable health care. Caregivers are encouraged to seek help and support from family, friends, and professional colleagues without hesitation.

The authors trust that these recommendations and suggestions will be helpful. By all means, try them. Those that work well, share with others. These suggestions will help to develop collaborative, mutually respectful arrangements with patients. In turn, these relationships will help the caregiver and patient to comprise a formidable team to fight the patient’s disease and promote his or her health.

Caring for “our fellow humans” is a wonderful and gratifying privilege. The medical profession enables individuals through a lifelong learning process to be competent caregivers. With this privilege comes tremendous responsibility. The authors trust this presentation of some of the best work of others presented in the form of guidelines, and a checklist will help physicians and caregivers to more effectively and comfortably provide equitable health care to more patients.

Finally, when there is good rapport with the patient, the clinician may encourage the patient to consider the risk and benefits of participation in a clinical trial, if asked.

## Footnotes

1 The term “patient” is used in this paper for simplicity’s sake. The terms client, resident, consumer, and member, among others, are also used to refer to the individual who is receiving clinical services.

2 Both immigrants and refugees are at risk of unequal treatment. The terms are often used interchangeably and are different: “An immigrant is someone who chooses to resettle to another country…. A refugee is someone who has been forced to flee his or her home country. As such, refugees can apply for asylum in the United States, a process that could take years.” CNN http://www.cnn.com/2014/07/15/us/immigrant-refugee-definition/

3 International Medical Interpretation Association (IMIA) Guide on Working with Medical Interpreters, http://www.imiaweb.org/uploads/pages/380_5.pdf.

4 An example of a useful video is: Communicating Effectively Through an Interpreter DVD, www.xculture.com.

5 Author’s note: some interpreters believe that serving as a cultural broker is a conflict of interest with their role as an interpreter. They believe that a separate individual, e.g., outreach worker, patient representative, should perform this role.
